# Supplementary material for: The content and completeness of women-held maternity documents before admission for labour: A mixed methods study in Banjul, The Gambia
Source: PLoS One. 2020 Mar 6;15(3):e0230063. doi: 10.1371/journal.pone.0230063 (PMC7059937; doi:10.1371/journal.pone.0230063)
Supplement: S1 Table — Antenatal care is provided as part of the Maternal, Child health and Family Planning program (MCHFP) and can take place at a variety of health facilities ranging from mobile health posts and local health centres to the tertiary hospital in Banjul. Some health centres have birth facilities, others only provide antenatal care and tell mothers to go to hospital to deliver. Primary healthcare centres can refer women to any of the three hospitals, normally the closest maternity unit geographically. Women experiencing complications in hospitals in provinces further inland (‘upcountry’) are sometimes referred to Hospitals 1 or 2. (DOCX) [file pone.0230063.s002.docx]

**Supporting Table 1:** Table of hospital background information

Antenatal care is provided as part of the Maternal, Child health and Family Planning program (MCHFP) and can take place at a variety of health facilities ranging from mobile health posts and local health centres to the tertiary hospital in Banjul. Some health centres have birth facilities, others only provide antenatal care and tell mothers to go to hospital to deliver. Primary healthcare centres can refer women to any of the three hospitals, normally the closest maternity unit geographically. Women experiencing complications in hospitals in provinces further inland (‘upcountry’) are sometimes referred to Hospitals 1 or 2.

| **Hospital 1** | **Hospital 2** | **Hospital 3** |
| --- | --- | --- |
| The only official tertiary hospital in The Gambia, providing specialist care and the largest numbers of doctors of any of the hospital in the Gambia. Offers specialist emergency obstetric care. | A growing hospital in the centre of the urban coastal area offering emergency obstetric care | A maternity specific facility in a more community setting offering emergency obstetric care |
| Highest referral rate | It is a referral centre for many of the surrounding health centres | Low referral rate |
| Receives the most complex cases | Still refers the most complex cases to Hospital 1 | Refers complex cases to Hospital 1 or 2 |
| 56 beds (8 on high dependency unit) | 28 beds (no high dependency unit) | 33 beds (10 on high dependency unit) |
| Doctors 33  Midwives 27  Nurses 29 | Doctors 5  Midwives 19  Nursing Attendants 12 Nurses 12 | Doctors 4  Midwives 12  Nurses 20 |
