# Supplementary material for: The content and completeness of women-held maternity documents before admission for labour: A mixed methods study in Banjul, The Gambia
Source: PLoS One. 2020 Mar 6;15(3):e0230063. doi: 10.1371/journal.pone.0230063 (PMC7059937; doi:10.1371/journal.pone.0230063)
Supplement: S2 Table — (DOCX) [file pone.0230063.s003.docx]

**Supporting Table 2:** Table of qualitative participant demographics

| **Characteristic** | **Categories** | **Number of Participants (%)** |
| --- | --- | --- |
| **Age** | 25 and under | 3 (10) |
|  | 26-35 | 17 (56.6) |
|  | 36-45 | 7 (23.3) |
|  | 46-55 | 2 (6.6) |
|  | 56 and over | 1 (3.3) |
| **Gender** | Male | 10 (33.3) |
|  | Female | 20 (66.6) |
| **Profession** | Doctor | 8 (26.6) |
|  | Midwife | 13 (43.3) |
|  | Nurse | 9 (30) |
| **Years of clinical experience** | 0-5 | 15 (50) |
|  | 6-10 | 5 (16.6) |
|  | 11-20 | 8 (26.6) |
|  | 21 and over | 2 (6.6) |
| **Religion** | Muslim | 25 (83.3) |
|  | Christian | 5 (16.6) |
| **Ethnic group/ tribe** | Mandinka | 10 (33.3) |
|  | Fula | 8 (26.6) |
|  | Wolof | 3 (10) |
|  | Manjago | 3 (10) |
|  | Aku | 2 (6.6) |
|  | Serahuli | 1 (3.3) |
|  | Serer | 1 (3.3) |
|  | Jola | 1 (3.3) |
|  | Tukulor | 1 (3.3) |
