# Supplementary material for: The content and completeness of women-held maternity documents before admission for labour: A mixed methods study in Banjul, The Gambia
Source: PLoS One. 2020 Mar 6;15(3):e0230063. doi: 10.1371/journal.pone.0230063 (PMC7059937; doi:10.1371/journal.pone.0230063)
Supplement: S3 Table — Answers were not mutually exclusive, respondents could select more than one option—therefore %s do not sum to 100. Answers are as patient described, unprompted. (DOCX) [file pone.0230063.s004.docx]

**Supporting Table 3: Reason for admission according to woman’s own description**

| **Symptom/reason for admission** | **Number, n=250 (% of cases)** |
| --- | --- |
| Abdominal pain | 208(83.2%) |
| Thought I was in labour | 161(64.4%) |
| My waters broke | 103(41.2%) |
| Vomiting | 59(23.6%) |
| PV bleed | 58(23.2%) |
| Headache / dizziness | 12(4.8%) |
| Regular testing | 7(2.8%) |
| Generally felt unwell | 5(2.0%) |
| Someone told me to come here | 4(1.6%) |
| Birth before arrival | 4(1.6%) |
| Planned C-section | 3(1.2%) |
| Collapse | 2(0.8%) |
| Other | 7(2.8%) |
|  |  |

Answers were not mutually exclusive, respondents could select more than one option - therefore %s do not sum to 100. Answers are as patient described, unprompted.
