# Supplementary material for: The content and completeness of women-held maternity documents before admission for labour: A mixed methods study in Banjul, The Gambia
Source: PLoS One. 2020 Mar 6;15(3):e0230063. doi: 10.1371/journal.pone.0230063 (PMC7059937; doi:10.1371/journal.pone.0230063)
Supplement: S1 Text — (DOCX) [file pone.0230063.s005.docx]

**Supporting Text 1:** Questionnaire

# Questionnaire; *version 1.1*

### Title: An investigation into the handover practices at admission to and discharge from maternity and obstetric services in Banjul, the Gambia.

| **PARTICIPANT STUDY NUMBER** |  |
| --- | --- |
| **DATE (DD/MM/YYYY)** |  |
| **TIME OF QUESTIONNAIRE (24h)** |  |
| **HOSPITAL NAME** |  |
| **RESEARCHER** |  |

Instruction to the researcher:

*Please remind patients that their data will be anonymised and stored safely and securely. They can withdraw or terminate the interview at any time and do not need to answer a question if they do not want to. Confirm that they have given consent and understand the purpose and procedure of the study.*

*Complete ‘Demographics’ and either/both ‘Admission’ or ‘Discharge’ sections as relevant to the specific participant. Please follow instructions and tick the appropriate box that matches the patient’s response or fill the appropriate space legibly. Return completed questionnaires to research supervisor with consent form*

**Is the participant currently taking part in any other MRC study?**

- Yes_1_
- No_2_

**Language of interview:**

- English_1_
- Mandinka _2_
- Wolof_3_
- Other_4_ ……………………………………………………

**Interpreter present?**

- Yes (please give initials)_1_ …………………………………
- No _2_

**Admission or Discharge?**

- Admission _1_
- Discharge_2_
- Both_3_

## Section A: Demographics (either Admission or Discharge)

1. **Age (years) (please select only one option)**

- Under 16_1_
- 16-20_2_
- 21-29_3_
- 30-40_4_
- 41 or over_5_
- Don’t know_6_

1. **Highest level of completed education (please select only one option)**

- Not gone to school_1_
- Did not complete primary_2_
- Primary_3_
- Junior Secondary_4_
- Senior Secondary_5_
- College_6_
- Vocational_7_
- University_8_
- Arabic_9_
- Other (please specify)_10_ …………………………………………………

1. **What tribe do you belong to? (please select only one option)**

- Mandinka_1_
- Fula_2_
- Wolof_3_
- Serahuli_4_
- Serer_5_
- Jola_6_
- Other (please specify)_7_ ……………………………………………..
- None_8_

1. **What kind of work do you do for your main occupation? (please select only one option)**

- House wife (only mark this if mother has no other job)_1_
- Farmer_2_
- Animal husbandry_3_
- Fishing and related activities_4_
- Retail trade (selling)_5_
- Civil servant_6_
- Healthcare work_7_
- Other (please specify)_8_ ………………………………………….

1. **How long did it take you to reach this hospital today? (please select only one option)**

- Within 1 hour_1_
- 1-4 hours_2_
- Over 4 hours_3_

1. **What type of transport did you use to get to the hospital? (please select only one option)**

- Walked_1_
- Donkey_2_
- Public transport (e.g. bus)_3_
- Motor vehicle (car/taxi/bus)_4_

1. **How many adults (17 years or older) live in the same house other than you? (please select only one option)**

- 0_1_
- 1-2_2_
- 3-4_3_
- 5-6_4_
- More than 6_5_

1. **How many children (16 years or younger) live in the same house other than you? (please select only one option)**

- 0_1_
- 1-2_2_
- 3-4_3_
- 5-6_4_
- More than 6_5_

1. **How many rooms do you have in your house for people to sleep in? (please select only one option)**

- 0_1_
- 1-2_2_
- 3-4_3_
- More than 4_4_

1. **How many (live) children do you have? (please select only one option)**

- 0_1_
- 1-2_2_
- 3-4_3_
- 5-6_4_
- More than 6_5_

1. **Where do you live? (please select only one option)**

- Banjul/Combo_1_
- Kanifing_2_
- West coast region (Kombo north, South, East and the Fonis)_3_
- Lower river region (Mansa Konko)_4_
- North Bank Region (Kerewan)_5_
- Central River Region (Janjanbureh)_6_
- Upper River Region (Basse)_7_

1. **What is the structure of your house? (please select only one option)**

- Brick/cement and tiled roof_1_
- Mud wall corrugated roof_2_
- Cement wall corrugated roof_3_
- Mud wall thatch roof_4_
- Other (please specify)_5_ ……………………………………………..

**Can you read and write? (please select only one option) – 12(b)**

- Yes_1_
- No_2_

## Section B (i): Admission

1. **What was the reason for your admission to the hospital? (please select only one option)**
   - I was referred by another health care professional_1_
   - I am self-admitted: had a ‘danger sign’ – e.g. vaginal bleeding, headache/dizzy_2_
   - I am self-admitted: felt labour pains/my waters broke_3_
   - Other (please specify)_4_

………………………………………………………………………

1. **Before coming to hospital, what was the first event/symptom that happened to make you want to see a health-worker/get medical attention? (please tick all that apply)**

- I thought I was in labour_1_
- My waters broke_2_
- I was in pain_3_
- I had vaginal bleeding_4_
- I was vomiting _5_
- I had a headache/felt dizzy_6_
- I generally didn’t feel well_7_
- My previous medical history (please specify)_8_

……………………………………………………………………………….

- Someone told me to see a medical professional_9_
- Routine tests_10_
- Planned Caesarean section _11_
- Other (please specify) _12_

……………………………………………………………………………….

1. **Have you visited anywhere to get advice before coming to hospital for this event/symptom? (please tick all that apply)**

- No, I came straight here when I thought there was a problem_1_ **(go to Q18)**
- This hospital outpatient department _2_
- Another hospital_3_
- Minor health centre_4_
- Outreach/trekking station_5_
- Traditional Healer _6_
- Pharmacist/Chemist_7_
- Other (please specify)_8_ …………………………………………………………………

1. **Who did you see at this contact? (tick all that apply)**

- Traditional Birth Attendant_1_
- Pharmacist_2_
- Village health worker_3_
- Community health nurse_4_
- Midwife_5_
- Hospital nurse_6_
- Doctor (obstetrician)_7_
- Other (please specify)_8_ ……………………………………………………………………

1. **Was it this contact that told you to come to hospital?**

- Yes_1_ **(if yes, please skip to question 19)**
- No_2_

1. **Who sent you to this hospital?**

- No-one told me, I decided to come here myself _1_
- This hospital outpatient department_2_
- Another hospital _3_
- Minor health centre – nurse/midwife/health-worker_4_
- Traditional birth attendant_5_
- Pharmacist/chemist_6_
- Family/friends_7_
- Other (please specify)_8_ ……………………………………………………………..

1. **Previous to this event/symptom, how many other contacts with health services have you had throughout this pregnancy? [please do not count traditional healers etc]**

- None, this is the first contact_1_ **(please skip to section C)**
- 1_2_
- 2_3_
- 3_4_
- More than 3_5_

1. **Prior to this admission what contact(s) have you had with health services? (please tick all that apply)**

- Antenatal clinic(s)_1_
- Scans/tests_2_
- Hospital admission_3_
- Outpatient appointment(s)_4_
- Home-visit_5_
- Consultation with Traditional Birth Attendant _6_

1. **Where did your most recent contact with a healthcare professional prior to this episode take place? (please tick only one)**
   - Home-visit_1_
   - Outreach station/trekking visit_2_
   - Minor health centre/static health facility _3_
   - Major health centre_4_
   - Another hospital_5_
   - This hospital_6_
   - No previous contact_7_
   - Other (please specify)_8_ ……………………………………………………………………………

## Section B (ii): Admission Documents

1. **Did you receive any paper documents (i.e. letter, antenatal card/document, test results, piece/scrap of paper) from any previous health service contacts to bring to this hospital (whether or not the doctor sent you here)? (please tick one only)**

- Yes, I have it with me_1_ **(Go to Q24)**
- Yes, I handed it to the doctors/nurses/midwives_2_ **(Go to Q24)**
- Yes, but neither I nor the staff have it_3_ **(Go to Q24)**
- No, I was given one but I did not bring it_4_ **(Go to Q23)**
- No, I was not given a document or note to bring_5_ **(end questionnaire)**
- No, I haven’t had a previous contact with a health service_6_

1. **If you had a paper document, but did not bring it today, please provide a reason why not? (please tick all that apply)**

- I forgot it at home_1_
- I lost it_2_
- I have always had it before but never used it so I did not bring it this time_3_
- I did not think that it was relevant to bring with me to hospital _4_
- I have never been asked for it here so I did not bring it this time _5_
- My children/spouse handle such documents, so I don’t know _6_
- Other (please specify)_7_

…………………………………………………………………………

1. **If you did bring a document, has a member of staff looked at it and used it? (please tick one only)**

- Yes_1_
- No_2_
- I don’t know _3_

1. **If you brought any document(s), can I please see it (them)? (please tick one only)**

- Yes_1_ **(please go to section D)**
- No _2_ **(please skip section D)**

Please look at patient notes or ask a member of staff:

1. **Reason for admission**

**…………………………………………………………………………………………………………………………..**

1. **Was the participant high-risk when admitted?**

***Defined: age <14, multiple pregnancy, severe anaemia, pre-eclampsia, PMHx (diabetes/heart condition), previous CX/obstruction***

- Yes_1_
- No_2_

## Section B (iii): FOR THE RESEARCHER – examine document(s) brought by the participant on *admission* to the ward

**Researcher, please ask to see any documents carried by the patient and check what type of document it is. NB please compare document to patient notes for accuracy**

| Antenatal card | Structured Referral Form | Prescription Card | Letter between HCPs | Discharge summary (date …………………..) | Scrap of paper | Other (please specify ………………………….) | Details present on admission document  (BOLD = minimal contents according to WHO) |
| --- | --- | --- | --- | --- | --- | --- | --- |
| □ | □ | □ | □ | □ | □ | □ | **1. Mother’s name** |
| □ | □ | □ | □ | □ | □ | □ | **2. Address** |
| □ | □ | □ | □ | □ | □ | □ | **3. Expected date of delivery/gestational age** |
| □ | □ | □ | □ | □ | □ | □ | **4. Parity** |
| □ | □ | □ | □ | □ | □ | □ | 5. Gravida |
| □ | □ | □ | □ | □ | □ | □ | **6. Relevant past medical history** |
| □ | □ | □ | □ | □ | □ | □ | **7. Relevant past obstetric complications** |
| □ | □ | □ | □ | □ | □ | □ | **8. Complications in antenatal period** |
| □ | □ | □ | □ | □ | □ | □ | **9. Problem referred for** |
| □ | □ | □ | □ | □ | □ | □ | **10. Tests/scans/treatments** |
| □ | □ | □ | □ | □ | □ | □ | **11. Tests/scans results** |
| □ | □ | □ | □ | □ | □ | □ | 12. Date |
| □ | □ | □ | □ | □ | □ | □ | 13. Menstrual History |
| □ | □ | □ | □ | □ | □ | □ | 14. Recommended place of delivery |
| □ | □ | □ | □ | □ | □ | □ | 15. Name of doctor/person who issued document/referred |
| □ | □ | □ | □ | □ | □ | □ | 16. HIV status |
| □ | □ | □ | □ | □ | □ | □ | 17. Detail is illegible |
| □ | □ | □ | □ | □ | □ | □ | 18. Document appears incomplete |
| □ | □ | □ | □ | □ | □ | □ | 19. emergency/high-risk |
| □ | □ | □ | □ | □ | □ | □ | 20. medications |
| □ | □ | □ | □ | □ | □ | □ | 21. Mother’s age |
| □ | □ | □ | □ | □ | □ | □ | 22. Family planning |

## Section C (i): Discharge

1. **Do you plan to see a healthcare provider for a check-up after you leave this hospital? (please tick all that apply)**

- Yes – postnatal clinic_1_
- Yes – hospital appointment_2_
- Yes – other (please specify)_3_ ………………………………………………………………
- No_4_ **(please skip to Q29)**

1. **If you do plan to see another healthcare provider, where will you visit? (please tick all that apply)**

- Another ward in the hospital_1_
- Another hospital_2_
- This hospital outpatient/post-natal clinic_3_
- Health centre_4_
- Static health facility antenatal clinic_5_
- Outreach/trekking station_6_
- Traditional Healer_7_
- Pharmacist/chemist_8_
- Other (please specify)_9_ …………………………………………………………………………..

1. **How will you tell them what has happened here in the hospital? (please tick all that apply)**

- The doctor/midwife/nurse has written on my antenatal card/postpartum document so I can show them that_1_
- The doctor/midwife/nurse has given me a letter/discharge summary to take with me_2_
- The doctor/midwife/nurse has told me and I have written it down on a piece of paper so I can tell staff I see in the future_3_
- The doctor/midwife/nurse told me what has happened (without me asking) so I can tell staff in the future_4_
- I asked the doctor/midwife/nurse to tell me so I can tell the other staff I will see in the future_5_
- I asked the doctor/midwife/nurse to tell my family member/friend who is with me and they will tell other staff I may see_6_
- The doctor/midwife/nurse is sending my information to the next health care staff I am seeing (e.g. via telephone/letter)_7_
- I do not know_8_
- Other (please specify)_9_ …………………………………………………………………..

1. **Please explain in as much detail as you can what you know about what has happened to you during your stay and what you expect to now happen when you are discharged. (please select only one option)**

Researcher should compare patient response to any discharge note or medical records and judge if the patient has correctly understood. If no note is present, ask staff on ward for an explanation and see if it is the similar to the patients’.

- Patient understands almost all things_1_
- Patient broadly understands events_2_
- Patient only understands the basic events of her stay_3_
- Patient understands very little_4_

1. **Do you think it is important to get a written explanation of what has happened to you at the hospital and what you should do when you are discharged? (please tick all that apply)**

- **yes** – why?
- I do not know_1_
- It helps me when I attend post-natal services _2_
- I can get attended to faster when I next see a health professional_3_
- It feels more professional_4_
- I feel more confident about what to do next_5_
- Other (please briefly specify)_6_

………………………………………………………………………………………………………

- If **no** – Why?
- I do not know_1_
- The note gets lost_2_
- Everyone receives the same care regardless of notes or documents_3_
- Other (please specify) _4_

………………………………………………………………………………………………………

1. **If you have had a document given to you by the hospital to take with you, please may I see it? (please select only one option)**

- Yes_1_
- No _2_

1. **Did the participant experience any ‘critical conditions’?**

- Yes_1_
- No_2_

**…………………………………………………………………………………………………………………………………..**

## Section C (ii): FOR THE RESEARCHER – examine document(s) brought by the participant on *discharge* from the ward

| Structured Discharge Form | Structured Referral Form | Prescription Card | Letter between HCPs | Antenatal Card | Scrap of paper | Other (please specify ………………………….) | Details present on discharge document  (BOLD = minimal contents) |
| --- | --- | --- | --- | --- | --- | --- | --- |
| □ | □ | □ | □ | □ | □ | □ | 1. Date (of discharge) |
| □ | □ | □ | □ | □ | □ | □ | 2. Mother’s name |
| □ | □ | □ | □ | □ | □ | □ | 3. Date and time of delivery |
| □ | □ | □ | □ | □ | □ | □ | 4. Parity |
| □ | □ | □ | □ | □ | □ | □ | 5. Gravida |
| □ | □ | □ | □ | □ | □ | □ | 6. Relevant obstetric history |
| □ | □ | □ | □ | □ | □ | □ | 7. Mode of delivery (Caesarian/forceps/vacuum/induced) |
| □ | □ | □ | □ | □ | □ | □ | 8. Complications/details of birth (e.g. episiotomy) |
| □ | □ | □ | □ | □ | □ | □ | 9. Birth defects/syndromes |
| □ | □ | □ | □ | □ | □ | □ | 10. Blood Loss |
| □ | □ | □ | □ | □ | □ | □ | 11. Apgar score |
| □ | □ | □ | □ | □ | □ | □ | 12. Baby’s gender |
| □ | □ | □ | □ | □ | □ | □ | 13. Birth weight |
| □ | □ | □ | □ | □ | □ | □ | 14. Place of birth |
| □ | □ | □ | □ | □ | □ | □ | 15. Name of staff who delivered |
| □ | □ | □ | □ | □ | □ | □ | 16. Name of doctor/person who issued document |
| □ | □ | □ | □ | □ | □ | □ | 17. Tests/scans/treatment |
| □ | □ | □ | □ | □ | □ | □ | 18. Tests/scans/treatment results |
| □ | □ | □ | □ | □ | □ | □ | 19. HIV status |
| □ | □ | □ | □ | □ | □ | □ | 20. Contraception |
| □ | □ | □ | □ | □ | □ | □ | 21. Vaccination |
| □ | □ | □ | □ | □ | □ | □ | *22. Detail is illegible* |
| □ | □ | □ | □ | □ | □ | □ | *23. Document appears incomplete* |
| □ | □ | □ | □ | □ | □ | □ | 24. Mother’s age |
| □ | □ | □ | □ | □ | □ | □ | 25. Address |
| □ | □ | □ | □ | □ | □ | □ | 26. Date of next appointment |
| □ | □ | □ | □ | □ | □ | □ | 27. Location of next appointment |
| □ | □ | □ | □ | □ | □ | □ | 28. Medications |

**Researcher, please ask to see any documents carried by the patient and check what type of document it is. NB please compare document to patient notes for accuracy**
