# Supplementary material for: The content and completeness of women-held maternity documents before admission for labour: A mixed methods study in Banjul, The Gambia
Source: PLoS One. 2020 Mar 6;15(3):e0230063. doi: 10.1371/journal.pone.0230063 (PMC7059937; doi:10.1371/journal.pone.0230063)
Supplement: S3 Text — (DOCX) [file pone.0230063.s007.docx]

**Supporting Text 3:** Definitions of ‘High-risk’ and ‘Complications’

High-risk:

- Pre-eclampsia/pregnancy induced hypertension
- Multiple pregnancy
- Previous C-section
- Previous obstructed labour
- Past medical history of heart conditions or diabetes
- Severe anaemia
- Age <14 years

Complicated delivery includes (not an exhaustive list):

- any delivery not defined as spontaneous vaginal delivery
- pre-eclampsia
- obstruction
- placenta praevia
- placental abruption
- post-partum haemorrhage
- still-birth
- Intra-uterine fetal death
- Fetal distress
- Uterine rupture
- Premature rupture of membranes (PROM)
- Severe anaemia
