# Supplementary material for: The content and completeness of women-held maternity documents before admission for labour: A mixed methods study in Banjul, The Gambia
Source: PLoS One. 2020 Mar 6;15(3):e0230063. doi: 10.1371/journal.pone.0230063 (PMC7059937; doi:10.1371/journal.pone.0230063)
Supplement: Attachment — Submitted filename: Responses to Reviewers.docx [file pone.0230063.s008.docx]

23^rd^ October 2019

Dear Plos ONE editors,

We thank the reviewers for their kind comments on the manuscript and have edited the document to address their concerns. Specific responses to reviewers’ individual comments can be found in the table below.

We have also addressed the recommendation to format the document in line with PlosONE recommendations and have labelled files appropriately.

| **Comment Number** | **Reviewer** | **Comment** | **Official Response** |
| --- | --- | --- | --- |
| 1 | 1 | This study is well written and very specific to people in field. one of few research that merge quantitative and qualitative data. | Thank you, we are glad that the benefits of merging quantitative and qualitative data are seen.  Maternity care is a high priority for the world health organization and sustainable development goals (as part of SDG 3). Although the sub-topic of maternity cards is specific, they are widely used, especially in low-middle income countries. Furthermore, they have featured in WHO guidelines both in the past and as recent as 2018, despite most of the evidence for them being ‘low-certainty’. Maternity cards are the cornerstone of antenatal care and delivery in Low-Middle income countries due to their facilitation of handover (which is key to patient safety across all specialties and countries). We hope that this study, although specific to the Gambia and maternal health, will be relevant to other low-income countries (LICs) and countries in the region that face similar challenges, especially as there is a limited amount of evidence regarding maternity cards from LICs. The effectiveness and feasibility of the card and some of the problems we identified could be generalised to a much wider number of countries where such research is yet to be done. |
| 2 | 2 | Isn’t the women-held maternity documents already well established? If so, the study should focus on either the feasibility in a local setting or completeness/accuracy of the records but not the efficacy in general. I think the authors already took these points into account as reflected by the design, results, and discussion. Hence, some of the texts (e.g. Line 59-61), and the title, should be revised at the authors’ discretion. | We agree with this comment that we have not conducted an efficacy study. Therefore have made changes in the wording in our ‘aims’ and throughout the paper to emphasise that the study explores the completeness of documents in a real-world setting. We have also made changes to the title to make this clearer. |
| 3 | 2 | The qualitative results were too brief and did not integrate the quantitative findings as proposed. The strength of the mixed methods design was not utilized as anticipated. | We understand that we have missed an opportunity to really make the most of the mixed-methods design. Therefore we have made multiple changes.   1) The qualitative analysis was reviewed and more supporting quotations and subthemes were added to the qualitative results table 2) Qualitative results that support the quantitative findings were more clearly signposted in the discussion. Furthermore, some of the qualitative findings were relevant in explaining the results of the rerun of the multivariate analysis of the quantitative data. 3) Qualitative results were used to help select clinically important variables for the rerun of the logistic regression which has been noted in the methodology section 4) Qualitative literature found in a new literature search and from the WHO 2018 Evidence Review of maternity documents was added to the background. |
| 4 | 3 | The topic of maternity cards is an interesting and important one and I applaud the authors for their work. I strongly support mixed methods manuscripts; however, this one tries to cover too much ground, and unfortunately, the quality of the text and analyses were sacrificed as a result. I have concerns about the quality of this study and the manuscript itself, which I describe in detail below. I suggest that the coauthors regroup and decide on a narrower focus of a potential future manuscript. As a side note, in a future manuscript, I suggest incorporating language around how maternity cards can be empowering to women in taking care of themselves and advocating for their care. | Thank you very much in acknowledging the importance of this topic. As mentioned above in response to comment 3, we realise we have not made the most of the mixed-methods design and so have worked on multiple ways of integrating the findings (see above).  We recognise that much of the ‘too much ground’ partly stems from lack of integration of the quantitative and qualitative findings. It was also a product of finding that the situation on the ground was far more complex than simply the use of the maternity card, and synthesis of the results regarding ‘other documentation’ and ‘referral sheets’ presented challenges. Through significant reorganisation of tables, renewed analysis and added evidence to the qualitative findings, we have tried to substantially strengthen the foundations of our results.  Specifically, Table 1 and 2 have been combined and reorganized into the three hospitals rather than referred/not referred. We have converted one of the tables into a visual chart. We have moved the table describing reasons for admission to supplementary materials. We have removed the table discussing ‘missing data’ which confused the interpretation of the results. We have rerun the logistic regression analysis and revised the table accordingly.  Throughout the revised manuscript we have tried to put focus and emphasis on the fact that the paper is about the completeness of the documents as for the documents to have the desired improved effects on care, they have to be complete.  Our background section now tries to acknowledge that there is a good existing evidence base for women themselves appreciating the maternity card for the ‘empowerment’ and involvement in their own care. A previous and renewed literature search revealed that the major gap in research was partly on the feasibility of the card and its completeness in a (especially low-income) setting. We have added an explanation that resource and time restraints meant that it was not possible to conduct interviews with women, which might have revealed themes regarding empowerment that were not identified through just HCP interviews. We recognise that the women have an important opinion to voice, but this would be an important future research possibility (and was actually a feature in our concurrent research regarding discharge documentation). The combination of the good existing evidence base, lack of time and resources, and desire to keep the study focused on completeness of the documents, meant that we were not able to incorporate the impact of maternity cards on the women’s sense of empowerment. |
| 5 | 3 | The background section does not adequately cover what is already known about the use of maternity cards. The authors describe this as an efficacy study, but it more closely aligns with an effectiveness study since it takes place in a real-world setting. | We agree that this is certainly not an efficacy study. We have changed the title and references to the study type throughout the manuscript accordingly.  We have expanded our background section to include parts of our literature search, and multiple references, that we were previously required to remove due to work count limits and limits to the number of references allowed for our original submission to PLOS Medicine. Therefore, we hope the background section now adequately covers what is known about the maternity cards. Notably, since the original literature search, a ‘WHO Review of Evidence’ has been published regarding women-held documents in maternity care and this has been commented upon heavily in the new background section and discussion. |
| 6 | 3 | The authors do not mention the sampling procedure for women participating in the quantitative proportion of the study. Did researchers interview each woman who was discharged until they reached a certain number of women on that day? On line 162, the authors write that research days were selected “at random and including weekends.” The authors fail to describe the specific procedure for randomization. Ultimately, the methods section needs to be written so that these findings could be reproduced. | We have made appropriate changes to our methodology section to describe more accurately our sampling procedure.  We attempted to purposively select an even distribution of weekdays and weekends across the three study sites to increase the likelihood of obtaining a representative sample. We then recruited as many women being discharged on that day as possible. We did so by being present on the ward from 9 until 1 as we had information from staff that that was the time period that discharges took place. We then asked the women to wait for us to speak to them before they left. Unfortunately we do not know exactly how many women were missed each day, although we can comment on the total number of discharged and that we approximately sampled a quarter of all discharges from the three hospitals in the period. |
| 7 | 3 | Further, on line 204, the authors mention that participant were assigned to FGDs or IDIs solely based on their availability. This raises a concern as these two qualitative methodologies have very distinct and differing purposes. For example, FGDs are often used to understand group norms or group dynamics while an IDI allows for a private, in-depth conversation. | Initially, we planned semi-structured interviews (SSIs) to obtain in-depth views and experiences which may be difficult to elicit in a group setting and focus group discussions (FGDs) to explore more in-depth group dynamics, agreed behaviours and ways of operation. However, the FGDs proved difficult to arrange due to staff availability; it was seemingly rare for large numbers of staff to be available at the same time. For the SSIs, sampling was much more systematic. Participants were allocated to either FGDs or one-to-one SSIs; no staff participated in both.  ^This above paragraph has been included in the qualitative methodology section. |
| 8 | 3 | Additionally, I would want to know why women were not interviewed to understand their perspectives of the maternity card process. If it was a matter of resources, that is fine, but it would have been good to acknowledge that in the text. | As has been mentioned above (response 6), because the study objectives were mainly around the feasibility and completeness of the documents, it was considered that it would be more valuable to interview HCPs about the use of the cards and potential reasons why the cards would not be completed accurately. Only HCPs write in the cards and so completeness depends on the HCP not the woman. Additionally, time and resource constraints played a part in the decision. We have added a paragraph to explain this decision in the qualitative methodology section and have added mention of how this would be important for future study in the discussion. |
| 9 | 3 | There are too many tables - with much more data than what is covered in the text. There are often not adequate text descriptions of the data in the tables.  The discussion and conclusion sections are broad and would need to go deeper with the implications of the research as well as recommendations. | The tables have been significantly restructured and more description of the tables has been added as footnotes.  Specifically, Table 1 and 2 have been combined and reorganized into the three hospitals rather than referred/not referred. We have converted one of the tables into a visual chart. We have moved the table describing reasons for admission to supplementary materials. We have removed the table discussing ‘missing data’ which confused the interpretation of the results. We have rerun the logistic regression analysis and revised the table accordingly.  The discussion section has been expanded to include more of the qualitative findings and to more explicitly explore the findings of the logistic regression analysis. We have tried to emphasise the clear recommendations of the study – to make simple changes to the existing card, standardise the referral documents, include all essential information on the maternity card, and introduce national level guidelines – that we feel are best supported by our evidence. |
| 10 | 3 | Statistical analyses have not been performed appropriately and rigorously. In the presentation of descriptive statistics, there were often differing denominators and it was not always clear why. Confidence intervals were erroneously included when presenting descriptive statistics. For multivariate analyses, adjusted odds ratios were not included. Additionally, the authors do not control for clustering of the hospitals. | We have sought further expert statistical advice from two statisticians and efforts have been made to review the presentation of our results. The denominators should now be clearer, and if they have changed due to missing data points, this is now explicitly mentioned in the text or footnotes of the tables.  Confidence intervals have been removed from the presentation of descriptive statistics, as there inclusion was indeed erroneous.  The results of both unadjusted and adjusted odds ratios from the logistic regression analysis are presented in table 3. The way in which variables were selected for inclusion in the logistic regression has been carefully described in the re-written methods section.  As there were only three study sites, ‘hospital’ was included as a fixed predictor variable in the final model to control for its influence. |
| 11 | 3 | The language in the manuscript is not always intelligible or well organized. Additionally, the presentation of numbers and percentages are not consistent, which is quite distracting. | Alongside significant restructuring of the tables and the addition of a bar-chart, we hope that the presentation of our data is clearer. We have added descriptions and footnotes to the tables to explain the data in a clearer manner. Significant changes to the text in all sections of our paper have been made and we have tried to make improvements to the clarity of language used. As we have removed the erroneous presentation of confidence intervals in the descriptive data, and have reviewed the denominators, we hope that the numbers are now clearer. We now consistently present a % and n/N for every piece of data stated in the results section. |
| 12 | 4 | This is a very well-written paper describing interesting, and alarming, results of a mixed-methods study regarding woman-held documents in maternity care of the Gambia. The abundance of data is logically organized and intelligible, and results highlight specific areas for improvement in the completion of cards and, in turn, potential reductions in maternal mortality for the region. The authors conclusions are sound and suggestions for inclusion of space for risk-status and expected delivery date on maternity cards are fitting of the data and very reasonable recommendations for improving maternal care. However, there are aspects of the methods and discussion that detract from the current impact of this paper. Specifically, the methods used to develop the model require more detail to support their proper interpretation. A clearer description of why variables were included in the regression models is needed. | We have sought further expert statistical advice and the regression analysis has been re-run. Characteristics, in the new analysis, were chosen based on clinical rationale, supported by both literature and the findings from the qualitative arm of the study. If variables were found to be too closely related on examination of cross-tabulations and the correlation matrices resulting in multi-collinearity, then they were excluded and clinical rationale was used to choose the most appropriate variable. This process has been explained in the methods section.  The results of the new regression analysis have been explored far more extensively in the discussion and we linked them to the qualitative findings and the literature to better understand these associations. |
| 13 | 4 | Additionally, the discussion should be expanded to better explain and situate the findings. While this may be the first study to assess completeness of cards, the authors should enrich the discussion with comparisons of their results on rates of women carrying documents and qualitative findings with research in other LMIC. | We have added clearer reference to relevant studies into women carrying documents in the second paragraph of the discussion (and we hope throughout). Other literature has also been used to help understand the new regression analysis results. |
| 14 | 4 (specifics) | Line 96: It is unclear why the authors refer to anecdotal evidence?? Is there not empirically-based data? A reference to anecdotal data does not belong in the abstract. | We agree with this comment and therefore have removed the sentence from the abstract. A WHO 2018 evidence review that suggests, based on key-informant data, that guidelines, training and support is needed to encourage appropriate use and we have mentioned this in the discussion. |
| 15 | 4 (specifics) | Line 123: LICs should be spelled out first (i.e. “low-income countries (LICs)”). | Thank you; this has been changed in line with your recommendation. |
| 16 | 4 (spec) | Line 136 should be changed to just LIC. | Thank you; this has been changed in line with your recommendation. |
| 17 | 4 (spec) | Line 127: Missing a “)” after (10) | Thank you; this has been changed in line with your recommendation. |
| 18 | 4 (spec) | Figure 1: If possible, please rotate the top image so it is easier to read. | Thank you; this has been changed in line with your recommendation. |
| 19 | 4 (spec) | Lines 193-197: The method by which variables were chosen to be included in regression analyses needs to be made clearer. Why were these specific few variables chosen? For example, what does distance from hospital have to do with completion of cards? What were the “appropriate exploratory models”?? | The regression analysis has been re-run. Characteristics were chosen based on clinical rationale, supported by both literature and the findings from the qualitative arm of the study. If variables were found to be too closely related following examination of cross-tabulations and the correlation matrices,resulting in multi-collinearity then they were excluded and clinical rationale was used to choose the most appropriate variable to include. This process has been explained in the methods section.  The methods now contains a clearer description of the statistical methods and “appropriate exploratory models” has been removed. |
| 20 | 4 (spec) | Table 2 should have asterisk, like Table 1, indicating which variables were included in the modeling. | Thank you; this has been changed in line with your recommendation. |
| 21 | 4 (spec) | Line 247: Shouldn’t this be 79/250 not 251? | Thank you; this has been changed in line with your recommendation. |
| 22 | 4 (spec) | Line 334-335: Please expand on the studies referenced here. Did they have similar findings to the present study? Why yes or no? | The discussion has been significantly restructured to include a more detailed literature review and explicit comparison of the results to the literature. |
| 23 | 4 (spec) | Lines 354-367: What about the other significant sociodemographic factors? What hypothesis do the authors have for the association between sociodemographics (being a housewife, living close to the hospital, literacy) and card completion? | The results of the new regression analysis (where variables were selected based on clinical rationale from the literature and the qualitative arm of the study) have been explored far more extensively in the discussion and we tried to link them to the qualitative findings and the literature to explain the potential reasons for them. |
| 24 | 4 (spec) | Lines 361-362: This appears to be the only reference to the qualitative data in the discussion. The authors should expand on how their findings are related to previous research and/or describe more the relation between their quant and qual data | We have re-written a lot of the discussion section with heavy emphasis now placed on reconciling the quantitative and qualitative findings to build a picture. We have especially made use of the rerun of the logistic regression analysis to relate our findings to previous literature.  We have also rerun our literature review, which helpfully highlighted studies we had not previously commented upon. |
| 25 | 4 (spec) | Line 362: I believe this should be changed to “in our sample, 100%..”. “In fact” seems to indicate that staff always review 100%, which cannot be inferred from this data. | Thank you; this point has been removed from the discussion as it was considered to not be clear. Greater discussion of the qualitative findings has replaced it. |
| 26 | 4 (spec) | Line 381: Please expand on in what ways you would anticipate the situation to be different in rural Gambia and why. | Following previous discussions we have had with colleagues in The Gambia alongside added evidence provided by key-informants in the most recent WHO review of evidence on maternity cards, we have tried to expand on how the situation may be different in rural Gambia in the discussion of the limitations of the study. We make particular parallels with a study in Pakistan.  *Since urban hospitals were sampled, the results may be less generalisable to rural Gambia than the urban Banjul area, although all rural areas around Banjul did refer women to these three hospitals. In Pakistan it was shown that maternity cards were more effective in rural than urban centres(40). On discussion with public health colleagues in the Gambia and reflecting on our study results that show women further from the hospitals had less complete documents, we would expect document use and completeness might be lower. This may be due to differing resource levels at the rural hospitals and clinics that might place more time-pressure on clinicians.* |
| 27 | 4 (spec) | Line 387: What type of sub-group analyses were under-powered? What analysis of high-risk women was underpowered? Almost half of the sample was high-risk. | Thank you; the sentence in question was in reference to analysis performed in previous drafts that was not included in the final publication. Therefore it has been removed. |
| 28 | 4 (spec) | Line 392: Mother should be changed to mothers. | Thank you; this has been changed in line with your recommendation. |
| 29 | PLOS | Please ensure that your manuscript meets PLOS ONE's style requirements, including those for file naming. The PLOS ONE style templates can be found at  [http://www.journals.plos.org/plosone/s/file?id=wjVg/PLOSOne_formatting_sample_main_body.pdf](http://www.journals.plos.org/plosone/s/file?id=wjVg/PLOSOne_formatting_sample_main_body.pdf) and [http://www.journals.plos.org/plosone/s/file?id=ba62/PLOSOne_formatting_sample_title_authors_affiliations.pdf](http://www.journals.plos.org/plosone/s/file?id=ba62/PLOSOne_formatting_sample_title_authors_affiliations.pdf) | We have altered the formatting of the document in line with the information in the links you have provided. We have separated the supplementary materials and figures from the file, and labeled them appropriately. |

We hope that these responses to the reviewers’ comments sufficiently answer their concerns. Please see our annotated and clean manuscript to see the changes we have made.

Yours sincerely,

Lotta Gustafsson and Fides Lu

[LTG469@student.bham.ac.uk](mailto:LTG469@student.bham.ac.uk)
