# Supplementary material for: The content and completeness of women-held maternity documents before admission for labour: A mixed methods study in Banjul, The Gambia
Source: PLoS One. 2020 Mar 6;15(3):e0230063. doi: 10.1371/journal.pone.0230063 (PMC7059937; doi:10.1371/journal.pone.0230063)
Supplement: Attachment — Submitted filename: Responses to reviewers.docx [file pone.0230063.s009.docx]

Dear Plos ONE editors,

We thank the reviewers again for their kind comments on the manuscript and have edited the document to address their concerns. Specific responses to reviewers’ individual comments can be found in the table below.

| Reviewer | Comment | Response |
| --- | --- | --- |
| 3 | I couldn't find S1 appendix and perhaps this information is already included there, but could the authors include the number of participants and the cadre of participants in the FGDs as they did for the SSIs in the body of the text? | The S1 appendix provides some information about each of the hospital study sites, including the total number of nurses, midwives and doctors working at each site. We will make sure that this appendix is uploaded successfully on our next submission.  To address your point we have tried to clarify the wording of this sentence/paragraph to make it clearer what cadre of participants were in each of the FDGs. |
|  | I have a few comments on table 1. The number of children includes 6 twice- "5-6" and "6 or more." Also, the number of previous contacts includes 3 twice- "1 to 3" and "3 or more." Additionally, I don't fully understand the difference between the "1d" and "2d" notations. Can the authors clarify the difference between these? Also, what does "2d2d" in the high-risk section mean? | Apologies, these were errors when reconfiguring the table from the SPSS data – it was meant to say “more than 6” and “more than 3”, thank you for spotting it.  Apologies, the “2d2d” was another formatting mistake and has been corrected. Hopefully this now makes the notation easier to understand.  The 1d and 2d shows where missing values were in each subgroup – i.e. in high-risk, two women from hospital 2 had a their risk status missing from their questionnaire sheet, therefore in the total column two women had this data point missing. In ‘number of previous contacts’, one participant from hospital 1 and one participant from hospital 2 had this piece of data missing from their questionnaire sheets, which totals two women missing this data (the far right column).  Due to these mistakes we went back over the table to ensure all data was correct and have made a few appropriate changes. We have also changed Supplementary Material 1 due to the mislabelling of the hospital numbers. |
|  | In the results section, lines 343-347, it would be useful to add odds ratios and specify what variables were significant in unadjusted and adjusted models. | Thank you – we have added these to the paragraph (please see below).  In logistic regression analysis, being literate in English (OR 2.04 [95% C.I. 1.08-3.85]), having 1-4 children compared to having fewer or more (OR 4.4 [95% C.I. 1.04-18.07]), having more than 3 contacts with healthcare during pregnancy (OR 2.16 [95% C.I. 1.15-4.03]) were all positively and significantly associated with minimum criteria fulfilment. Travelling further than 1 hour to get to hospital (OR 0.34 [95% C.I. 0.15-0.74]) and attending a hospital other than the tertiary referral centre (Hospital 2 OR 0.45 [95% C.I. 0.19-1.02], Hospital 2 OR 0.22 [95%C.I. 0.08-0.60]) were negatively associated with minimum criteria fulfilment [Table 3]. |
|  | In table 3, I'm not sure why/how the reference categories have p values. For example, for a categorical variable with 3 categories or levels, the p values would be looking at statistical significance comparing the 1st and 2nd categories/levels and the 1st and 3rd categories/levels, so there should only be p values for the 2nd and 3rd categories. | The p value against the reference category is the p value for the variable’s overall contribution to the model (as are the p values for binary variables). As the p values against other categories do not add information above that provided by the 95% Cis, they are redundant and have been removed from the table (but could be reinstated if the editor wishes). |
| 4 | Title – I believe you’d want to use a colon not semi-colon. | Thank you, we have now changed this in line with your recommendation. |
|  | Qualitative methods: Traditionally you would want more than one coder of transcripts. The authors should ex  plain why they only had one person code all transcripts and what was agreement on the 4 transcripts coded by other researcher? | It is considered good practice in qualitative research for more than one research to be involved in coding transcripts but there is no absolute requirement for all transcripts to be coded by another researcher (see for example N Gale et al BMC Res Methods 2013 where it is suggested that another researcher code four transcripts). The concept of interrater agreement is contested in qualitative research (C Pope BMJ 2000), and in this study the other coder contributed to the development of coding, improving the quality of the analysis. Time and resource precluded the double coding of all transcripts and a comment to that effect could be added if the editor wishes. |
|  | Table 1: • Ages seem to not include 20 (under 20 and 21+) • Something is wrong with the education row. The numbers don’t line up to categories and the last category is Islamic… • What is 2d 2d in missing high-risk? | - We have changed this to ’20 and under’, thank you for spotting this mistake. - The lining up of Table 1 when opened on a windows computer was flawed. We have now edited the table on a windows computer to make sure that the numbers line up. ‘Islamic’ category refers to home or small group based education about the Quran, and the student does not learn to read or write English which is the written language or commerce, business and general use. However as the person has exposure to some education we felt it is important to keep this category separate. We have inserted a foot note to say: renamed this as informal/formal Quranic education without learning to read English the national language - This was a typo and has been correcting, thank you for spotting it. |
|  | Line 269 - It is not clear what documents were considered in this calculation. The authors state they were “not including loose scan or test result sheets” but then seem to consider these as “less frequently presented documents (ultrasound reports, prescription notes, scraps of 272 paper, child health reports, miscellaneous lab requests/results)”. What is a loose scan vs an ultrasounds, for example? | Thank you very much for making us aware of this statement that we agree lacks clarity. We recognised that the calculation also features in our abstract, so we returned to our raw data to ensure that our statement was accurate. The phrase ‘loose sheets’ remained there after an earlier draft and needed review.  We decided that to ensure clarity, we have calculated the value as ‘antenatal cards or referral sheets’ and now explain this clearly both in the paragraph and the abstract. We have made this decision to not include ‘less frequently presented documents’ (which were often ‘loose’) for two reasons. Firstly, these were the only two documents to include significant clinical information (the less frequently presented documents were therefore considered less clinically important to comment on when considering if a woman had presented with any documentation). Secondly, there was a data collection error where 40 participants did not have the presence ultrasound/test requests recorded. This was alluded to on line 323 (“35 women who had attended scanning were not able to be assessed for ultrasound scan presence in their documentation” – 5/40 did not attend scanning). We have added explanation of this to line 324 to make it clear that it was researcher error.  With regards to the paragraph you have commented on, one participant presented with no documents, but we are missing data as to whether she brought an ultrasound sheet with her or not. Because of this we cannot comment on the number of women who brought ‘no documentation’ of any kind. If the editors feel further clarity is needed regarding this matter, please let us know. |
|  | Line 282 – Why couldn’t the content of the card be assessed? | The content of the cards could not be assessed because although the mother could confirm that she had brought an antenatal card with her to hospital, the cards themselves were elsewhere in the hospital e.g. on the Neonatal unit with the baby.  We have added this to the text. |
|  | Line 301 – Why couldn’t the content of the referral be assessed? | The content on some occasions could not be assessed because some of the referral sheets that women recalled bringing with them were elsewhere on the ward rather than with the woman/unable to found by researchers.  We have added this explanation to the text. |
|  | Figure 4 does not make sense to me. I don’t understand the y axis, did women have hundreds of documents? This does not seem the best way to illustrate the point made in the text, that only the maternity card had met all 9 criteria. | We have removed the numbered labels on the Y-axis to make the bars represent the percentage of that type of document that met the minimum criteria (either 9 out 9, or 8 out of 9). We have also altered the X-axis title to simplify the message.  The minimum criteria fulfilment of ‘referral sheets’ and ‘other documents’ has been added to emphasise that only the maternity card met all 9 criteria.  We are happy to adapt or remove this figure if the editor wishes. |
|  | Line 344 – Why don’t the authors mention the significant association of numbers of contacts throughout pregnancy in the results and discussion intro paragraph? | Thank you, this has now been added (with odds ratios) to the appropriate paragraphs. |
|  | Table 3. I am unsure of what the p-value next to the reference category would mean. What is this a comparison of? | Please see response to comment on Table 3 above. |
|  | Table 4. [B] and [F] unnecessary considering the bolded row headings of barrier and facilitator. | Thank you, we have changed this in line with your suggestion. |
|  | Line 452 - It seems that the qualitative data DOES support this (quote from M6 in org barriers). The authors might want to reflect on how this was an issue identified here, so perhaps training should emphasize how helpful clinicians find the cards as a motivator. Or, perhaps a different incentive to complete them is needed. | This was an important point to have been brought to our attention, thank you. We reflected on this and have added appropriate comments on this in our discussion. |
|  | Line 510 – ALMOST universally. | Thank you, we have made this change. |

We look forward to hearing from you.

Yours sincerely,

Lotta Gustafsson and Fides Lu
