# Supplementary material for: The content and completeness of women-held maternity documents before admission for labour: A mixed methods study in Banjul, The Gambia
Source: PLoS One. 2020 Mar 6;15(3):e0230063. doi: 10.1371/journal.pone.0230063 (PMC7059937; doi:10.1371/journal.pone.0230063)
Supplement: Attachment — Submitted filename: Responses to reviewers.docx [file pone.0230063.s010.docx]

Dear Plos ONE editors,

Thank you for your comment regarding Figure 4. We have removed figure 4 and added it to the supplementary materials.

We look forward to hearing from you.

Yours sincerely,

Lotta Gustafsson and Fides Lu
